# Learning Low-Rank Class-Specific Dictionary and Sparse Intra-Class Variant Dictionary for Face Recognition

**DOI:** 10.1371/journal.pone.0142403

**Published:** 2015-11-16

**Authors:** Xin Tang, Guo-can Feng, Xiao-xin Li, Jia-xin Cai

**Affiliations:** 1 School of Science, Huazhong Agricultural University, Wuhan, Hubei province, China; 2 School of Mathematics and Computational Science, Sun Yat-sen University, Guangzhou, Guangdong province, China; 3 College of Computer Science and Technology, Zhejiang University of Technology, Hangzhou, Zhejiang province, China; Jiangnan University, CHINA

## Abstract

Face recognition is challenging especially when the images from different persons are similar to each other due to variations in illumination, expression, and occlusion. If we have sufficient training images of each person which can span the facial variations of that person under testing conditions, sparse representation based classification (SRC) achieves very promising results. However, in many applications, face recognition often encounters the small sample size problem arising from the small number of available training images for each person. In this paper, we present a novel face recognition framework by utilizing low-rank and sparse error matrix decomposition, and sparse coding techniques (LRSE+SC). Firstly, the low-rank matrix recovery technique is applied to decompose the face images per class into a low-rank matrix and a sparse error matrix. The low-rank matrix of each individual is a class-specific dictionary and it captures the discriminative feature of this individual. The sparse error matrix represents the intra-class variations, such as illumination, expression changes. Secondly, we combine the low-rank part (representative basis) of each person into a supervised dictionary and integrate all the sparse error matrix of each individual into a within-individual variant dictionary which can be applied to represent the possible variations between the testing and training images. Then these two dictionaries are used to code the query image. The within-individual variant dictionary can be shared by all the subjects and only contribute to explain the lighting conditions, expressions, and occlusions of the query image rather than discrimination. At last, a reconstruction-based scheme is adopted for face recognition. Since the within-individual dictionary is introduced, LRSE+SC can handle the problem of the corrupted training data and the situation that not all subjects have enough samples for training. Experimental results show that our method achieves the state-of-the-art results on AR, FERET, FRGC and LFW databases.

## Introduction

**Fig 1:**
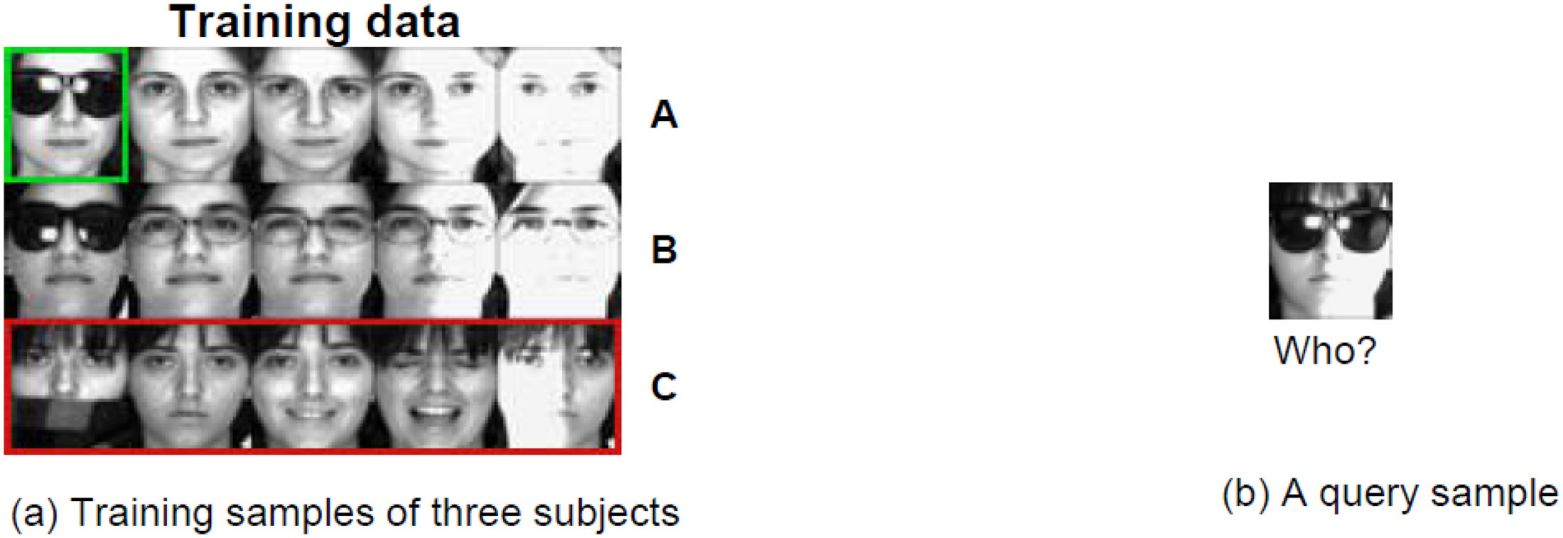
An example of face recognition with occlusions in the training set. (a) Training samples of three subjects. (b) A query image from Person C. The facial images are from AR database [38].

Face recognition has been an active topic in machine learning, computer vision and pattern recognition research due to its potential value for applications and theoretical challenges. Face recognition is widely applied in real life, such as online image search, law enforcement, access control for secure facilities (e.g., prisons and office buildings), security surveillance, and etc. Many face recognition approaches have been proposed, such as, Eigenfaces [1], Fisherfaces [2], Laplacianfaces [3], and shown to provide promising results in recent years. However, face recognition continues to be a challenging task owing to the variability in illumination, pose, expression, occlusion and the small sample size problem. For example, Fig 1 demonstrates an example of face recognition with occlusion. The training set contains images from three different persons. There is a query sample from Person C. However, since the disguise and light condition, this query image looks like the image bounded by green rectangle, which belongs to Person A. Therefore, various robust face recognition techniques have been developed to handle variations in illumination [4] and occlusion [5]. A new LDA-based face recognition method has been presented in order to solve the small sample size problem [6].

Recently, one of the hottest and most interesting methods for robust face recognition is sparse representation based classification (SRC) [7]. SRC is based on the assumption that the samples from each individual subject lie on a class-specific linear subspace. However, for a query image, the training samples of all subjects are collaboratively used to represent it. Therefore, SRC treats the training images of all subjects as a dictionary and finds the sparsest solution of a test image with respect to the dictionary. Obviously, dictionary quality is very important for sparse representation. The metaface learning of face images has been presented by Yang *et al*. [8], which learns a dictionary for each class individually. Learning discriminative dictionary has been proved to improve the performance of face recognition dramatically [9] [10] [8] [11] [12] [13]. Jiang *et al*. [14] propose a label consistent K-SVD algorithm to learn a discriminative dictionary, which associates label information with each dictionary item. A Fisher discrimination dictionary learning scheme [13] for sparse representation is developed, and it employs the Fisher discrimination criterion to learn class-specific dictionary for each subject independently. The performance of these methods deteriorates when there are sparse large noises in the training data, such as shadows and specularities, corruptions, and occlusions. In addition, these methods are problematic when the training set is unbalanced in sense that certain individuals have very few training samples compared to others.

The low-rank matrix recovery technique has been successfully applied to various fields, for instance multimedia [15], document analysis [16], salient object detection [17] and image processing [18]. One representative is robust principal component analysis (RPCA) [19], which decomposes a corrupted matrix into a sparse component and a low rank component. RPCA can be exactly solved via a nuclear norm regularized minimization problem. Considering that the training images are corrupted, low-rank matrix recovery has been used for denoising. Ma *et al*. [20] exploit rank minimization and propose a discriminative low-rank dictionary learning for sparse representation (DLRD_SR). DLRD_SR separates the noises in the training images by minimizing the rank of the sub-dictionary of each class. A low-rank matrix recovery algorithm with structural incoherent for robust face recognition has been presented by Chen *et al*. [21]. Chen’s method considers the noises in the training images and achieves well results when the training images are corrupted because of occlusion and disguise. A discriminative and reconstructive dictionary is constructed and a discriminative low-rank representation for image classification is obtained [22]. These algorithms separate the sparse noises from the training images and are robust to severe illumination variations or occlusions.

In practical scenarios, facial images contain uncertain and noisy information, such as illumination conditions, expression conditions, or occlusions. If given the training images of each subject which cover the facial variations of that person under testing conditions, face recognition will become an easy task. Obviously, this situation is not practical and face recognition is a small sample size problem in general [23]. However, the original SRC algorithm assumes that there is a sufficient number of training samples for each class. Therefore, Wagner *et al*. [24] extend SRC and introduce a method to obtain a set of training images of each subject for covering all possible illumination changes. In order to cope with the small sample size problem under the SRC framework, Extended SRC (ESRC) is proposed by Deng *et al*. [25], which utilizes images collected from external data to construct a intra-class variant dictionary. The variant dictionary is applied to represent the possible variations between the training and testing images. With the help of intra-class variant dictionary, ESRC outperforms SRC, especially when a single training image per class. However, there are two shortcomings in ESRC. Firstly, ESRC needs an external dataset and requires that the external data is very relevant to training and testing data, which may not be as readily available in real applications. On the other hand, images collected from external data might contain noisy, redundant, or undesirable information which would degrade the capability in covering intra-class variations [26]. Secondly, ESRC can’t deal with the cases where the training data are corrupted well. Given the training images of each class, ESRC and SRC don’t consider the difference between subject-specific feature, also known as the discriminative vector of each subject, and the intra-class variant feature. The intra-class variant feature capturing image-specific details, such as expression conditions, is non-discriminative and can be shared by all subjects. Fig 1 shows an example, in which one training image of Person A is occluded by a sunglass. SRC may treat the occluded region (sunglass) as the inherent feature of Person A and makes a wrong decision. For this reason, Mi *et al*. [27] have proposed two novel robust face recognition methods based on linear regression (RLRC 1 and 2). They consider that each class-specific subspace is spanned by two kinds of basis vectors. The first one is the common basis vectors shared by many classes; the other one is the class-specific basis vectors owned by one class only.

**Fig 2:**
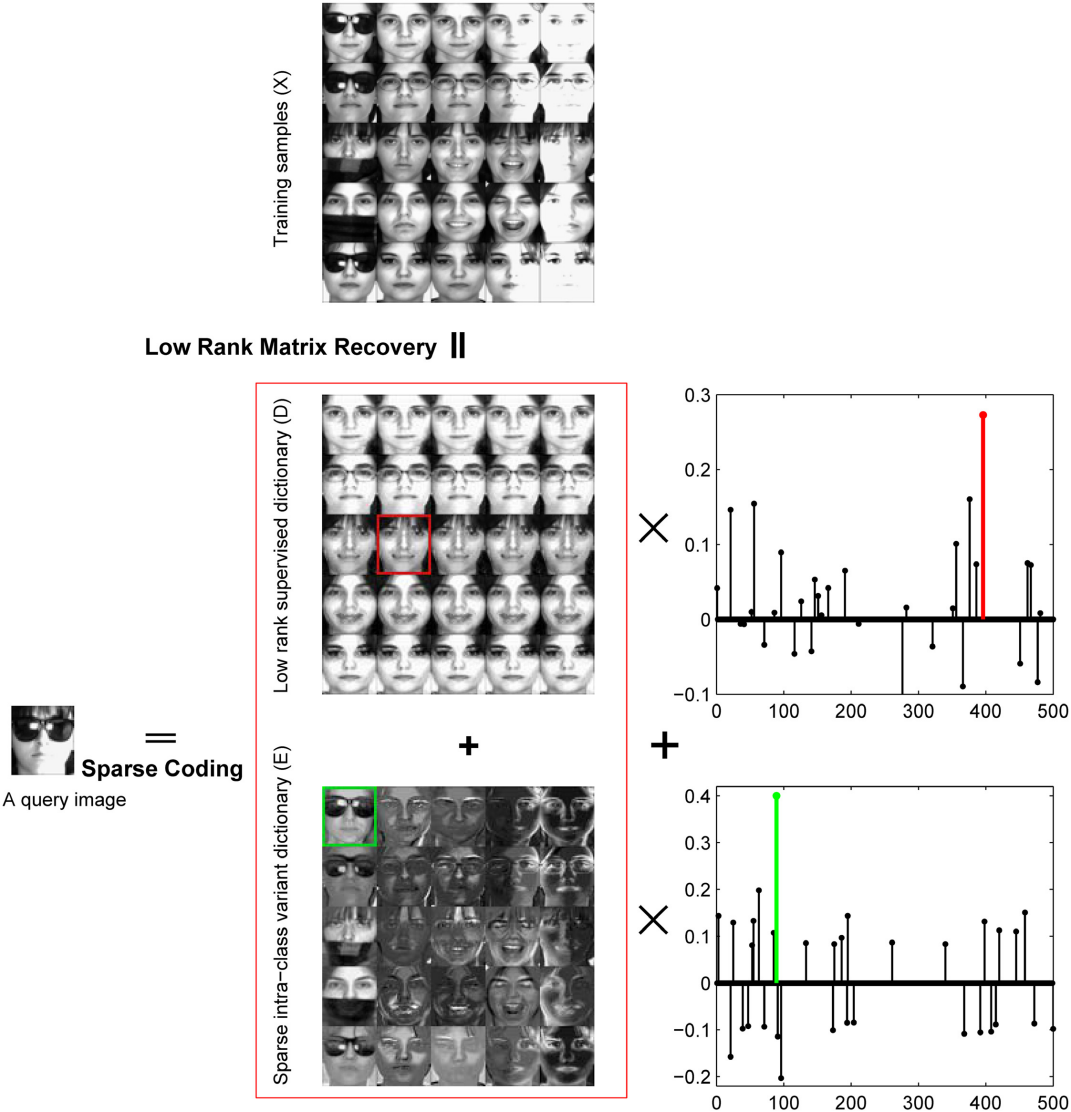
Motivation of LRSE+SC. The facial images are from AR database [38].

In this paper, we just consider face recognition from frontal views. Hence, the facial images of the same person often correlate with each other and if we stack the training images within the same subject into a matrix, this matrix should be approximately low-rank. To build a robust classifier against small sample size problem and the problem of unbalanced training set, we propose a novel face recognition framework by using low-rank and sparse matrix decomposition, sparse coding techniques (LRSE+SC). First, the training images of each individual are decomposed into a representation basis matrix of low rank and a sparse error matrix. The representation basis matrix determines the class-specific subspace. Many methods, for example DLRD_SR, ignore the interesting information contained in sparse large noises. The sparse error matrix, which represents the gross corruption of the training images, such as expression, occlusion, or illumination conditions, is very important for face recognition. It consists of the noise or within-individual variance and can explain why two images of the same subject do not look identical. Second, the representation basis matrix of all subjects are collected and the supervised dictionary is established. Meanwhile, we integrate the sparse error matrix of all subjects into a within-individual variant dictionary shared by all classes. We then combine the supervised dictionary with the within-individual variant dictionary to encode a query image with sparsity constraint. In this way, the class-specific dictionary differentiates the subjects and the within-individual variant dictionary is used to provide the essential reconstruction for the query image. Fig 2 presents the motivation of the proposed approach. Finally, as SRC, a reconstruction-based scheme for classification is adopted. From Fig 2, the query image is able to successfully recognized by our method. The experiments demonstrate that our method achieves very promising performance.

Three main contributions of this paper are as follows. Firstly, we decompose the training images of each class into a low-rank part and a sparse part by low-rank matrix recovery. The low-rank part is a representation basis matrix of each class and it determines the class-specific subspace. The class-specific dictionary captures the discriminative feature of each class and is owned by only one class. The sparse part accounts for intra-class variance and can be shared by other subjects. Hence, for each image, it can be decomposed into a vector from intra-class variant subspace and a discriminative vector from class-specific subspace. Secondly, we analyze the reason why SRC doesn’t work effectively when there are not enough training samples for each class. Thirdly, a supervised dictionary and a within-individual variant dictionary are builded to sparsely encode the query image. Our method is different from traditional dictionary learning methods (e.g. MFL, LCKSVD), which don’t consider the problem that not all individuals have plenty of training samples. The most important of our method is that we separate the within-individual variance information from the training images and introduce an auxiliary dictionary by using the sparse error matrix per class.

## Background

Firstly, let us present a typical face recognition problem. Assume there are *n* training images from *c* distinct classes, and *n*_*i*_ training images from the *i*th subject, *i* = 1, 2, …, *c*. Each image is represented as a vector *x*_*i*_*j*__ ∈ *R* ^*m*×1^, which means this image from the *i*th class. *m* denotes the dimension of feature space for all images. *X_i_* = [*x*_*i*_1__, *x*_*i*_2__, …, *x*_*i*_*n*_*i*___] ∈ *R* ^*m*×*n_i_*^ consists of training images belonging to the *i*th subject and *X* = [*X*_1_, *X*_2_, …, *X*_*c*_] ∈ *R* ^*m*×*n*^ denotes the training images matrix by concatenating all training samples. Given the training samples in *X*, the aim of face recognition is to classify a query image *y* ∈ *R* ^*m*^.

Recent research has proved that linear regression based algorithms, e.g. Nearest Feature Subspace (NFS) [28], Linear Regression Classification (LRC) [29], and Sparse Representation Classification (SRC), are extremely easy to use and powerful for face recognition. The linear regression based algorithms assume that the images of one individual subject lie on a class-specific linear subspace.

Sparse representation has attracted broad interest in various domains due to its great success in image processing. The basic idea of sparse representation is to represent a given test sample correctly with as few training samples as possible. SRC assumes that training samples from each subject lie on a linear subspace spanned by the training images from the given subject. Therefore, for a test sample *y* ∈ *R* ^*m*^ belonging to class *i*, if given sufficient training samples of class *i*, we have

$$\begin{array}{c} y=x_{i_{1}}\beta_{i_{1}}+x_{i_{2}}\beta_{i_{2}}+\cdots +x_{i_{n_{i}}}\beta_{i_{n_{i}}}=X_{i}\beta_{i}, \end{array}$$ (1)

where *β_i_* = [*β*_*i*_1__; *β*_*i*_2__; …; *β*_*i*_*n*_*i*___] ∈ *R* ^*n*_*i*_^ is the coefficient vector corresponding to *X*_*i*_. Of course, this class-specific subspace is embedded in the linear space spanned by all the training images. Hence, *y* can be cast as the linear combination of all training samples, *i.e*.

$$\begin{array}{c} y=X\beta , \end{array}$$ (2)

where *β* ∈ *R* ^*n*^ is a coefficient vector. Actually, we don’t know which class the query image *y* comes from. Hence, the goal of sparse representation is to represent *y* using as few training images as possible, which is computed by solving the following minimization problem:

$$\begin{array}{c} \begin{array}{cc} \hat{\beta } & =\arg\min_{\beta }{∥\beta ∥}_{0}\\ & {s.t.\;∥y-X\beta ∥}_{2}^{2}\leq \epsilon , \end{array} \end{array}$$ (3)

where *ϵ* is a pre-specified small constant and ∥⋅∥_0_ means *ℓ*_0_-norm. The above problem is NP-hard and it can be solved by approximating the *ℓ*_0_-norm with *ℓ*_1_-norm based convex relaxation. Hence, problem Eq (3) can be transformed to minimizing the reconstruction error with a *ℓ*_1_-norm regularizer, *i.e*.

$$\begin{array}{c} \hat{\beta }=\arg\min_{\beta }∥y-{X\beta ∥}_{2}^{2}+{\lambda ∥\beta ∥}_{1}, \end{array}$$ (4)

where *λ* is a scalar constant. In the ideal case, the entries of $\hat{\beta }$ are zeros except those associated with the column of *X* from the *i*th class. In practice, this is not real, the recovered coefficient vector $\hat{\beta }$ has most of its non-zero entries corresponding to the atoms belonging to the ground-truth class of the query image, while a few non-zero values are distributed elsewhere. Therefore, the query image *y* is assigned to the class which has the minimum reconstruction residual.

For SRC, the original training images act as a dictionary to represent the query image. Because the original face images may contain some noise, uncertain, or redundant information that can be negative to recognition, learning a dictionary from training images becomes an active topic. Yang *et al*. [8] propose a metaface learning (MFL) algorithm to represent the query image by a series of dictionaries learnt from each class. In order to achieve good performance, many discriminative dictionary learning methods are presented [9] [10] [11] [12] [13]. However, these dictionary learning methods need sufficient number of training samples per class.

## Proposed Method

In practice, training images which are corrupted (*i.e*., occlusions, lighting variations, facial expressions) violate the linear subspace assumption. Furthermore, due to insufficient number of training images, the query images of the *i*th class may be not lie on the subspace spanned by training images *X*_*i*_. Therefore, the performance of SRC will deteriorate in these two situations. In order to handle the small sample size problem, leave-one-class-out subspace model is proposed [27]. The leave-one-class-out subspace of each class consists of all the common vectors and class-specific basis vectors for other classes but does not include any class-specific basis vectors for itself. In this section, we will propose a novel face recognition framework by utilizing low-rank and sparse error matrix decomposition, and sparse coding techniques. Unlike leave-one-class-out subspace model, our method can explicitly extract class-specific basis vectors owned by only one class and separate within-individual variant basis vectors from the original training images.

### Basic Assumption

**Fig 3:**
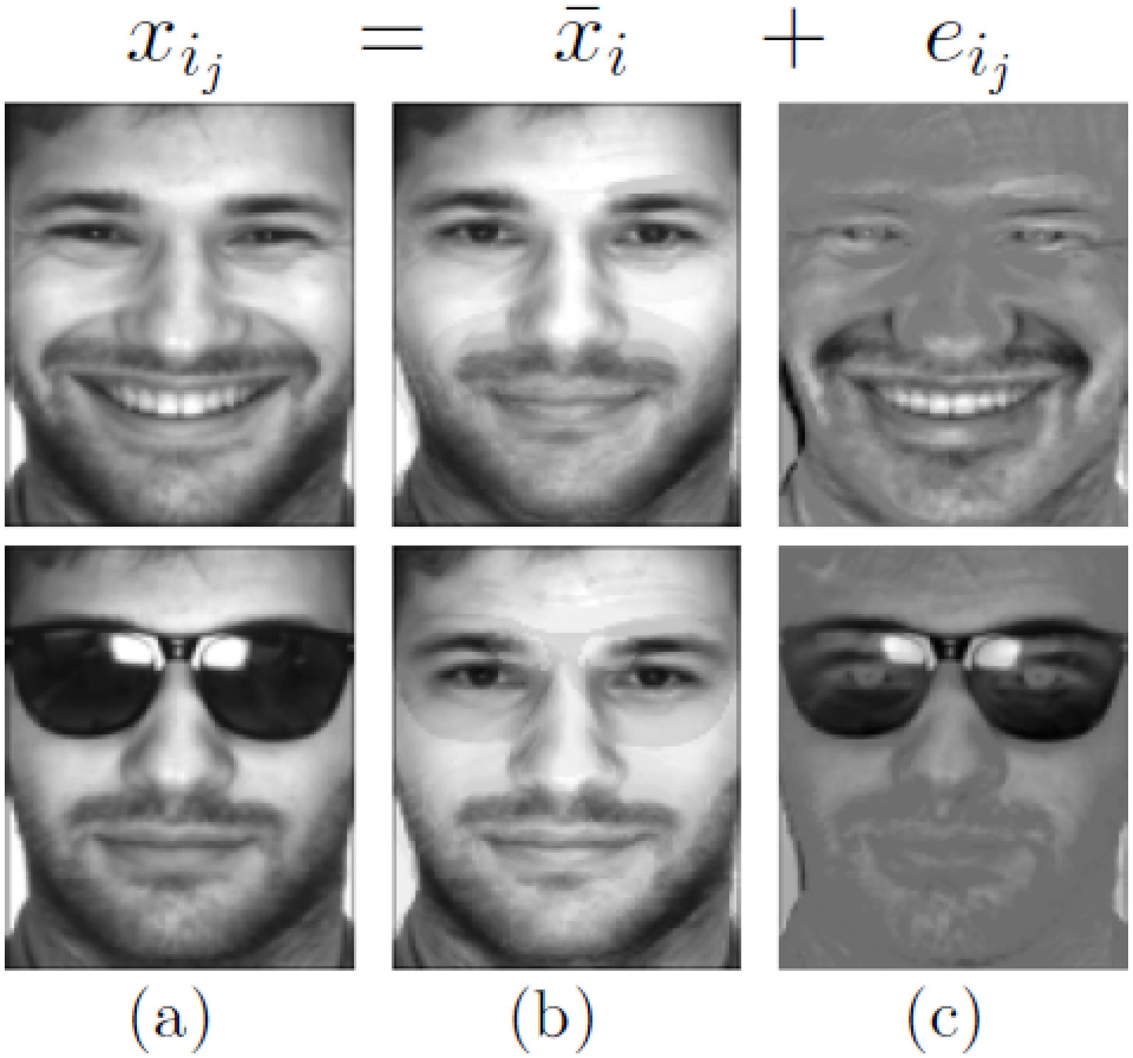
Facial component separation. (a) Original face images. (b) Neutral face images. (c) The sparse images corresponding to expression, or occlusion. The facial images are from AR database [38].

In this paper, we do not consider the impact of variations in pose and age. Because images are affected by variability in illumination, expression and occlusion, images of the same individual do not look identical to each other. We assume that *x*_*i*_*j*__ comes from the *i*th individual and can be represented as

$$\begin{array}{c} \begin{array}{c} x_{i_{j}}={\bar{x}}_{i}+e_{i_{j}}, \end{array} \end{array}$$ (5)

where ${\bar{x}}_{i}$ is the clean and neutral image of the *i*th individual, and the term *e*_*i*_*j*__ consists of noise or within-individual variance and is sparse. *e*_*i*_*j*__ may contain the information about illumination conditions, expression conditions, and even occlusions in the image *x*_*i*_*j*__. That is, a facial image can be decomposed into a neutral component and a sparse component pertaining to details on the face such as expressions, or occlusions (see Fig 3). Under this assumption, for another image *x*_*i*_*k*__(*j* ≠ *k*) of the *i*th subject, the difference between *e*_*i*_*j*__ and *e*_*i*_*k*__ can explain why two images (*x*_*i*_*j*__ and *x*_*i*_*k*__) both belong to the *i*th subject but do not look identical. On the other hand, two images from different subjects may have the same within-individual variance *e*. For example, in Fig 1, the query image looks like the training image with sunglass from Person A. Hence, many methods classify the query image as Person A due to the sunglass. If the sunglass is separated from the query image, we may make a right decision. For a image *x*_*i*_*j*__, it can be decomposed into signal ${\bar{x}}_{i}$ and noise component *e*_*i*_*j*__. ${\bar{x}}_{i}$ captures the structured patterns of the *i*th subject and thus it can be used for classification, while the within-individual variance *e*_*i*_*j*__ only contributes the essential representation for the image *x*_*i*_*j*__.

### Face Recognition by Using Low-rank Matrix Recovery and Sparse Coding Techniques

Wright *et al*. choose training samples as dictionary for sparse coding. If training images are corrupted, SRC fails to extract the class-specific feature of each subject from the original training images and can’t handle the cases when the training set is corrupted. For example, in Fig 1, the training image of Person C is occluded by scarf, the occluded regions (the scarf) might be regarded as the structure pattern of Person C. According to Eq (5), there exist the common patterns and the within-individual variance among images of the same class. The variability caused by the unbalanced lighting changes, variable expressions, and occlusions, can be shared by many subjects. On the other hand, SRC requires a large number of training samples of each subject to span the complete class-specific subspace. In this paper, we try our best to mitigate the negative effects of specific variance and utilize it.

Recently, low-rank matrix decomposition technique has received significant attention. As we know, principal component analysis (PCA) has been widely used for extracting low-dimensional information from the high-dimensional data. However, classical PCA lacks of robustness to grossly corrupted observations [30]. In order to robustify PCA, many approaches have been proposed in the literatures [31] [32] [19]. In particular, Wright *et al*. [19] recently have proposed a robust PCA method which is a powerful tool for various applications, such as image processing [18]. The training images which we have collected are often affected by expression, pose, occlusion or illumination. For dictionary learning methods, the dictionary learned from the original images might contain information about the image-specific details, such as expressions, occlusions. It has a negative effect on classification. The facial images from the same subject are correlated with each other and natural high-dimensional data often lies on a low-dimensional linear subspace. Meanwhile, each image contains image-specific details such as specularities and cast shadows, or noise with sparse support in the image. Therefore, the training images within each subject are decomposed into a low-rank matrix and a sparse matrix by using low-rank matrix recovery technique. The sparse matrix means that the images within each class undergo gross corruption such as occlusion, pose, or illumination changes.

For the noisy training images of the *i*th class, according to Eq (5), it can be modeled as:

$$\begin{array}{c} \begin{array}{c} X_{i}=D_{i}+E_{i}, \end{array} \end{array}$$ (6)

where each column of *D*_*i*_ represents the neutral image of the *i*th subject and *E*_*i*_ is the noise matrix of the *i*th class (*i* ∈ {1, 2, …, *c*}). Because the neutral images of each subject are correlate with each other, *D*_*i*_ is a low-rank matrix. *E*_*i*_ represents expressions, occlusions, specularities and cast shadows in the training images of the *i*th individual and is a sparse matrix. Therefore, this decomposition can be solved by the following optimization problem:

$$\begin{array}{c} \begin{array}{cc} \min_{D_{i},E_{i}} & rank\left(D_{i}\right)+\lambda ∥E_{i}{∥}_{0}\\ & s.t.\;X_{i}=D_{i}+E_{i}, \end{array} \end{array}$$ (7)

where ∥⋅∥_0_ represents the *ℓ*_0_-norm (the nonzero entries in the matrix) and *λ* is the parameter that trades off the rank term and the sparsity term. However, Eq (7) is non-convex and NP-hard to solve. Wright et al. [19] indicate that under broad conditions the aforementioned low-rank matrix recovery problem Eq (7) can be exactly solved via the following convex optimization problem:

$$\begin{array}{c} \begin{array}{cc} \min_{D_{i},E_{i}} & ∥D_{i}{∥}_{*}+\lambda ∥E_{i}{∥}_{1}\\ & s.t.\;X_{i}=D_{i}+E_{i}, \end{array} \end{array}$$ (8)

where the nuclear norm ∥*D*_*i*_∥_*_ approximates the rank of *D*_*i*_. To solve the optimization problem Eq (8), the augmented lagrange multiplier method proposed by Lin et al [33] can be adopted.

After the low-rank matrix *D*_*i*_ and sparse error matrix *E*_*i*_ for each subject have been learned, we denote *D* = [*D*_1_, *D*_2_, …, *D*_*c*_] and *E* = [*E*_1_, *E*_2_, …, *E*_*c*_]. *D*_*i*_ contains the structured patterns and discriminative feature of the *i*th subject. Therefore, *D*_*i*_ has a better representative ability than the original data *X*_*i*_ in describing the face images of the *i*th subject [21]. The class-specific sub-dictionaries (*D*_*i*_) of all subjects are combined to build the supervised dictionary *D*. On the other hand, the non-class-specific dictionary *E* only contributes to essential representation of the images, such as expression and illumination conditions, rather than discrimination. Since dictionary *E* represents non-class-specific variations, the random noises need to be decreased. This is done by removing dictionary atoms whose norm is less than an arbitrary-chosen threshold (e.g. 10^−3^).

According to the basic assumption, a query image *y* can be represented as

$$\begin{array}{c} y=\bar{x}+e=D\alpha +E\beta =\left[D\;E\right]\;\left[\begin{array}{c} \alpha \\ \beta \end{array}\right], \end{array}$$ (9)

where $\bar{x}$ is the natural image and it can be represented by *Dα*. Sparse error matrix *E* usually represents lighting changes, exaggerated expressions, or occlusions. *e* (*Eβ*) represents the image details of *y*, such as expression conditions or noise with sparse support in the image. We can use the within-individual variant dictionary *E* and supervised dictionary *D* to represent *y*. If there are redundant and over-complete facial variant bases in *E*, the combination coefficients in *β* are naturally sparse. Hence, the sparse representation *α* and *β* can be recovered simultaneously by *ℓ*_1_-norm minimization.

Based on Eqs (6) and (9), we propose a face recognition framework by using low-rank and sparse error matrix decomposition and sparse coding techniques (LRSE+SC). Our method treats the face recognition problem as finding a sparse coding of the query image in term of the supervised dictionary as well as the within-individual variant dictionary.

After introducing the two phases of the proposed method, the main steps of LRSE+SC are summarized in Algorithm 1.

**Algorithm 1** Low-rank matrix recovery and sparse coding for face recognition (LRSE+SC)

**Inputs:** A matrix of training images *X* = [*X*_1_, *X*_2_, …, *X*_*c*_] ∈ *R* ^*m*×*n*^ for *c* subjects and the query image *y*, parameters *λ*_1_, *λ*_2_.

**Output:** Class label of the query image *y*.

**Step 1**: Learning class-specific dictionary and intra-class variant dictionary by low-rank matrix recovery.

**for** *i* = 1: *c* **do**

  min_*D*_*i*_,*E*_*i*__ ∥*D*_*i*_∥_*_ + *λ*_1_∥*E*_*i*_∥_1_ *s*.*t*.*X*_*i*_ = *D*_*i*_ + *E*_*i*_

**end for**

**Step 2**: Building supervised dictionary and Within-individual variant dictionary.

 The supervised dictionary *D* = [*D*_1_, *D*_2_, …, *D*_*c*_] is make up of the class-specific sub-dictionaries *D*_*i*_. The within-individual variant dictionary *E* = [*E*_1_, *E*_2_, …, *E*_*c*_] is builded by integrate all the sparse error matrix of each subject. However, for each column of *E*, if the norm is less than a threshold *η* (e.g. *η* = 10^−3^), it is removed from *E*.

**Step 3**: Finding sparse representation of the query image *y* in term of new dictionary [*D* *E*].

 Solving the *ℓ*_1_-norm minimization problem

$[\begin{array}{c} \hat{\alpha }\\ \hat{\beta } \end{array}]=\arg\min_{\alpha ,\beta }∥y-\left[D\;E\right]\;[\begin{array}{c} \alpha \\ \beta \end{array}]{∥}_{2}^{2}+\lambda_{2}∥[\begin{array}{c} \alpha \\ \beta \end{array}]{∥}_{1}$.

**Step 4**: Classification

**for** *i* = 1: *c* **do**

$e\left(i\right)=∥y-D\delta_{i}\left(\hat{\alpha }\right)-E\hat{\beta }{∥}_{2}^{2}$

**end for**

*Identity*(*y*) = argmin_*i*_ *e*(*i*)

## Analysis of the Proposed Method

In this section, the justification of our method and the difference with SRC will be discussed. Linear regression based algorithms assume that the images from a single subject lie on a linear subspace. We denote the subspace spanned by the training images from the *i*th subject as *S*_*i*_. Thus, we have

$$\begin{array}{c} S_{i}=span\left\{x_{i_{1}},x_{i_{2}},...,x_{i_{n_{i}}}\right\}. \end{array}$$ (10)

Given a query image *y*, we assume that it comes from the *i*th class. If we have observed a sufficient number of training images per subject, then the query image can be well reconstructed by the training images belonging to the ground-truth class of it. Therefore, *y* ∈ *S*_*i*_. Obviously, SRC has achieved very promising results in this situation.

In practice, facial images might suffer from expression, illumination variations and even occlusions, there isn’t adequate number of training images for the *i*th class to cover the variations of the test image *y*, i.e., *y* ∉ *S*_*i*_. However, for large scale face recognition problems where the training sets contain large number of subjects, some training images from other subjects can be used to describe the test image *y*. Therefore, in this paper, we suppose that the test sample lies on the subspace spanned by all training samples, i.e., *y* ∈ *S* = *span*{*X*_1_, …, *X*_*c*_} and *y* ∉ *S*_*i*_. For SRC, the training samples, which are the most similar with the query image *y*, are selected to represent *y*. Since *y* ∉ *S*_*i*_, there exist training samples $x_{1}^{\prime },..,x_{p}^{\prime }$ which come from other classes and they can be used to represent *y*. Certainly, *y* can’t be modeled accurately by training images just from others because there must be some unique patterns owned by the *i*th class. So, we have

$$\begin{array}{c} y\in span\left\{x_{i_{1}},...,x_{i_{n_{i}}},x_{1}^{\prime },...,x_{p}^{\prime }\right\}\;and\;y\notin span\left\{X_{-i}\right\}, \end{array}$$ (11)

where *X*_−*i*_ represents all training samples except for the *i*th subject. According to Eq (11), the linear representation of *y* can be written as:

$$\begin{array}{c} y=\sum_{j=1}^{n_{i}}\alpha_{i_{j}}x_{i_{j}}+\sum_{j=1}^{p}\beta_{j}x_{j}^{\prime }, \end{array}$$ (12)

where *α*_*i*_*j*__ and *β*_*j*_ are the coefficients. Obviously, *α* = [*α*_*i*_1__; *α*_*i*_2__; …; *α*_*i*_*n*_*i*___] ≠ 0 and *β* = [*β*_1_; *β*_2_; …, *β*_*p*_] ≠ 0. Without loss of generality, assume that $x_{1}^{\prime },..,x_{p}^{\prime }$ all come from the *k*th (*k* ≠ *i*) subject. If the contribution of training data belonging to the *i*th class is small, it is possible that $∥\sum_{j=1}^{n_{i}}\alpha_{i_{j}}x_{i_{j}}{∥}_{2}<{∥\sum_{j=1}^{p}\beta_{j}x_{j}^{\prime }∥}_{2}$. Then SRC may classify *y* as the *k*th class. For example, the query image in Fig 1. Due to the sunglass, it looks like the image bounded by green rectangle and SRC recognizes it as Person A. In fact, the training samples of others are used to represent the regions of the test image *y* which might be caused by illuminations, expressions, or occlusions. However, SRC can’t separate these components (such as, illumination, expression variations) from the original training samples, hence, SRC treats them as discriminative feature of each subject.

According to the theories of linear subspace and Eq (5), *S*_*i*_, i.e., the subspace of Subject *i*, can be modeled as

$$\begin{array}{c} S_{i}=span\left\{x_{i_{1}}-{\bar{x}}_{i},x_{i_{2}}-{\bar{x}}_{i},...,x_{i_{n_{i}}}-{\bar{x}}_{i},{\bar{x}}_{i}\right\}=span\left\{e_{i_{1}},e_{i_{2}},...,e_{i_{n_{i}}},{\bar{x}}_{i}\right\}. \end{array}$$ (13)

In Eq (5), only one vector ${\bar{x}}_{i}$ is used to represent the class-specific information of images for Subject *i* and it is the discriminative component of this subject, while *e*_*i*_1__, *e*_*i*_2__, …, *e*_*i*_*n*_*i*___ are basis vectors for the within-individual subspace and explain why training images of Subject *i* do not look identical. Hence, the basis vectors of subspace *S*_*i*_ can be divided into two categories: The first one is the discriminative vector for each class; the other one is within-individual variant vector. However, in practical scenarios, facial images are affected by many factors, it’s not appropriate to describe the class-specific information of Subject *i* by just one basis ${\bar{x}}_{i}$. From Eq (6), utilizing low-rank matrix recovery technique, we can use a matrix *D*_*i*_ as representation basis matrix of Subject *i* and *S*_*i*_ is the subset of space spanned by *D*_*i*_ and *E*_*i*_, i.e.,

$$\begin{array}{c} S_{i}=span\left\{d_{i_{1}}+e_{i_{1}},d_{i_{2}}+e_{i_{2}},...,d_{i_{n_{i}}}+e_{i_{n_{i}}}\right\}\subset span\left\{d_{i_{1}},d_{i_{2}},...,d_{i_{n_{i}}}\right\}+span\left\{e_{i_{1}},e_{i_{2}},...,e_{i_{n_{i}}}\right\}, \end{array}$$ (14)

where *d*_*i*_*j*__ and *e*_*i*_*j*__ represent the *j*th (*j* = 1, …, *n*_*i*_) column of *D*_*i*_ and *E*_*i*_, respectively. We denote $S_{i}^{\prime }=span\left\{d_{i_{1}},d_{i_{2}},...,d_{i_{n_{i}}}\right\}$. Therefore, $S_{i}^{\prime }$ is a class-specific subspace and *span*{*e*_*i*_1__, *e*_*i*_2__, …, *e*_*i*_*n*_*i*___} represents the noise or within-individual variance of *X*_*i*_. We combine the sparse error matrices of all subjects into a within-individual variant dictionary *E* = [*e*_1_1__, …, *e*_1_*n*_1___, …, *e*_*c*_*n*_*c*___], which can be used to model the intra-class variations lighting conditions, expressions or occlusions. Therefore, *W* = *span*{*e*_1_1__, …, *e*_1_*n*_1___, …, *e*_*c*_*n*_*c*___} is a within-individual subspace. From Eq (14), we have $S_{i}\subset S_{i}^{\prime }+W$. For a query image from the *i*th subject, it lies in the linear subspace $S_{i}^{\prime }+W$. Therefore, the query image *y* in Eq (12) can’t be represented by training samples from Subject *i*, but it may be lie in $S_{i}^{\prime }+W$. Fig 2 shows an example. The query image *y* belongs to Subject 84. However, *y* ∉ *S*_84_ due to the occluded regions. By taking advantage of low rank matrix recovery, we obtain the within-individual subspace *W*. The query image *y* can be represented by the class-specific subspace $S_{84}^{\prime }$ and within-individual subspace *W*. Hence, LRSE+SC can alleviate the small sample size problem and the problem of the corrupted data.

From Eq (8), when *λ*_1_ tends to infinity, all the atoms of within-individual dictionary *E* are zeros. In this situation, our method LRSE+SC is equivalent to SRC.

## Results

In this section, several experiments are implemented to demonstrate the effectiveness of the proposed LRSE+SC algorithm by comparing it with the state-of-art on the AR, FERET, FRGC and LFW databases. Besides SRC, we compare our method with linear regression for classification (LRC) [29], Extended SRC (ESRC) [25], MFL, RLRC1 and RLRC2 [27]. In ESRC, we construct the intra-class variant dictionary by subtracting the class centroid of images from the same class. As we known, many algorithms [34] [35] [36] [37] can solve the *ℓ*_1_-regularized least squares problem. The feature-sign search algorithm is very fast and achieves high performance [35]. For fair comparisons, both LRSE+SC, SRC and ESRC use the feature-sign search algorithm to solve the *ℓ*_1_ minimization problem. The regularization parameters in all algorithms are tuned by experience. The Matlab code of LRSE+SC algorithm can be downloaded from http://www.researchgate.net/publication/264556568_LRAESC?ev=prf_pub.

### AR Database

The AR face database is employed because it’s one of the very few including natural occlusions. The AR database consists of over 4,000 frontal images for 126 individuals [38]. For each individual, 26 images are taken under different variations, including illumination, expression, and facial occlusion in two different sessions. All the images are cropped with dimension 44 × 40 and converted to gray scale. In this paper, we select a subset of 50 male subjects and 50 female subjects for our experiments (as [7] do). For each individual, there are six occluded images and the remaining seven are simply with illumination and expression variations in each session.

#### A. Face Recognition Without Occlusion

In this part, we just consider face recognition without occlusion, and the occluded face images aren’t considered. Hence, for each class, fourteen images with only illumination and expression changes are used for experiments.

**Fig 4:**
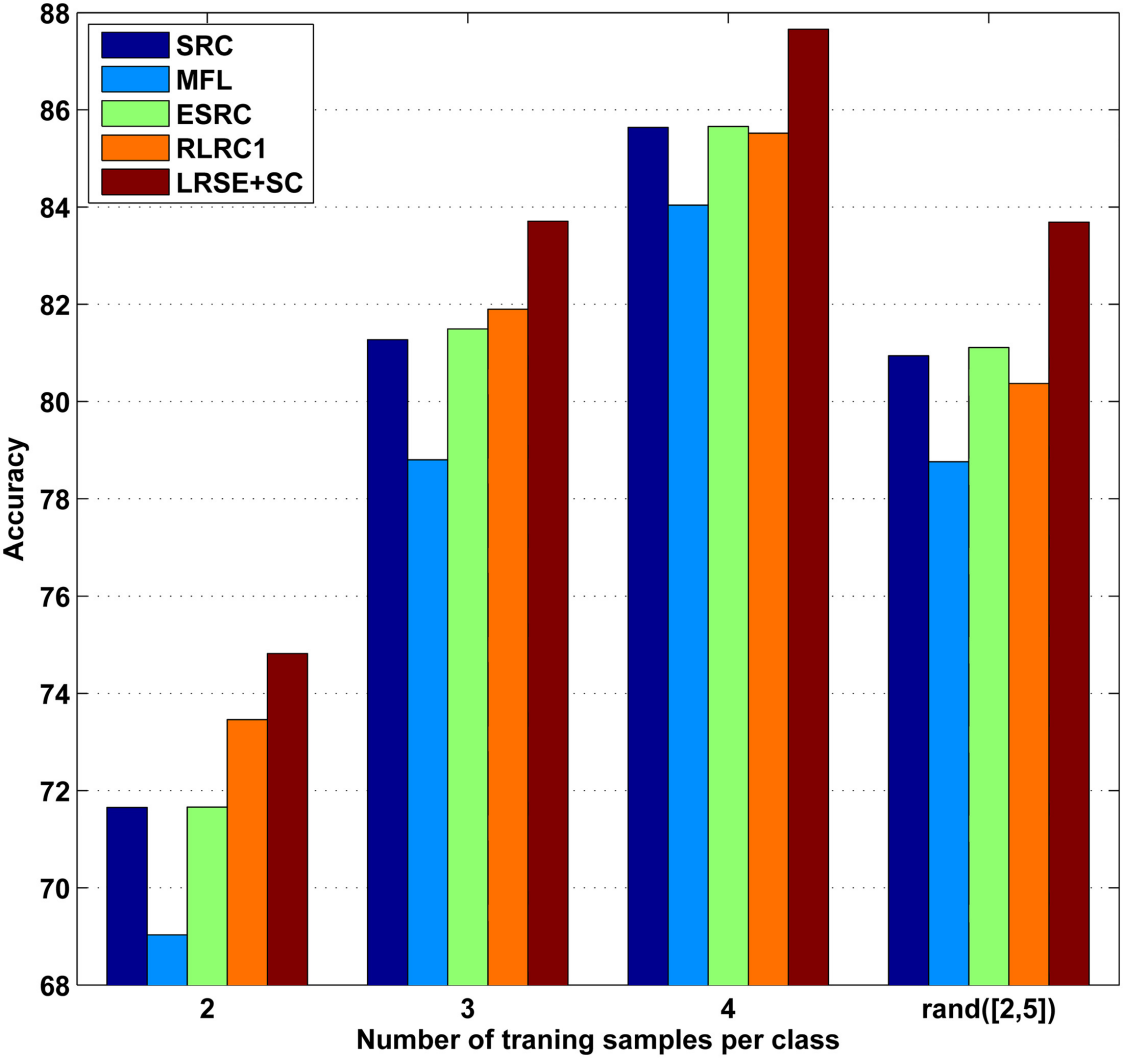
Recognition rates on AR database with different number of training samples per class. In this experiment, for each subject, 14 images with only illumination and expression changes are selected. *rand*([2, 5]) means the number of training images belonging to the *i*th class is a random number between 2 and 5.

In the first experiment, *n*_*i*_ images per class are randomly selected from Session 1 for training and the rest (14 − *n*_*i*_ per class) are used as query samples. This partition produce is repeated for 5 times and we compute the average results. *n*_*i*_ denotes the number of training samples of the *i*th class and it may be different for each class. To test undersampled effect, the number of training samples per class *n*_*i*_ is small. We set *n*_*i*_ = 2, 3, 4, *rand*([2, 5]) respectively, where *rand*([2, 5]) means that the number of training samples per class is a random number between 2 and 5. Hence, for each class, it’s obvious that there are insufficient training samples to span the variations of expression and illumination under testing conditions. The average recognition rates are demonstrated in Fig 4. Since the training data size is small, the recognition rates of all methods are poor. Compared with other methods, the recognition rates of LRC, RLRC2 are unacceptable and aren’t enumerated in Fig 4. For example, when *n*_*i*_ = 2, the classification accuracies of LRC, RLRC2, SRC, MFL, ESRC, RLRC1 and LRSE+SC are 36.52%, 45.15%, 71.65%, 69.03%, 71.66%, 73.46%, 74.82%, respectively. For all methods, the recognition rates rise as the number of training samples increases. As can be seen, our algorithm LRSE+SC outperforms all the other methods. When the number of training samples per each class is unequal (*n*_*i*_ = *rand*([2, 5])), the performance of LRSE+SC improves significantly. For example, the recognition rate of MFL is 78.76% while LRSE+SC achieves 83.69%.

In the foregoing experiment, there is not enough training data for each class. For the second experiment, we consider the scenario that there are sufficient training samples for some subjects. Hence, we randomly choose *p* classes ({*i*_1_, *i*_2_, …, *i*_*p*_}) and for each of these *p* classes, seven images with illumination and expression changes at Session 1 are selected for training. On the other hand, for the remaining classes ({*j*_1_, *j*_2_, …, *j*_100−*p*_}), only one image of each class at Session 1 is randomly selected and used as training sample. For each subject, seven images (without occlusion) from Session 2 are used for testing. In other words, for any class from {*i*_1_, *i*_2_, …, *i*_*p*_}, sufficient training samples are used for training, meanwhile, for others, there is only one training sample. We repeat this procedure for 5 times and report average recognition accuracy.

**Fig 5:**
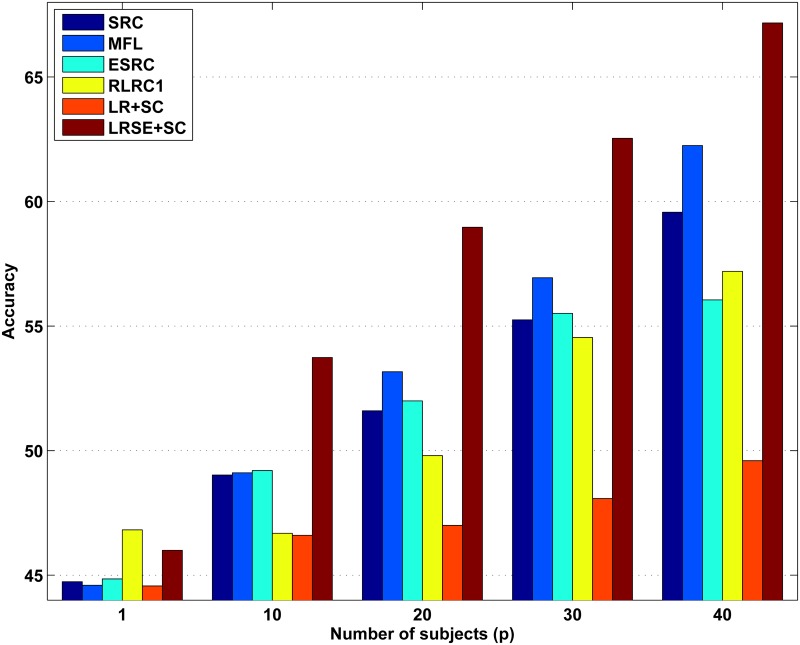
Recognition rates on AR database versus number of subjects (*p*). In this experiment, for each subject, 14 images with only illumination change and expression are selected. We randomly select *p* classes and for these subjects, all seven images at session 1 are used as training samples.

**Fig 6:**
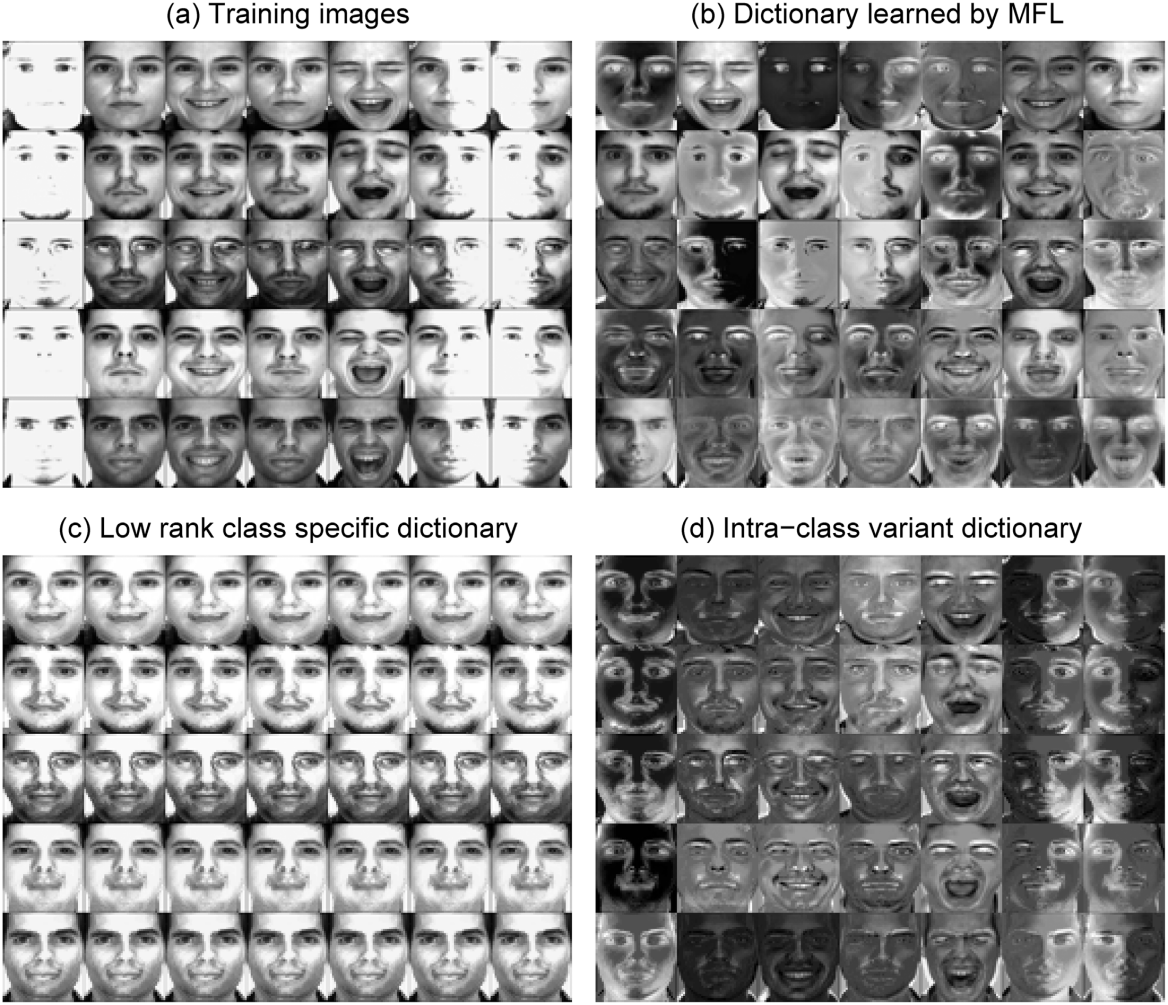
Dictionaries are learned by MFL, LRSE. (a) The training images of five persons. (b) Dictionary learned by MFL for the five persons. (c) Low rank class-specific dictionary (low rank matrix) learned by LRSE for each person. (d) Intra-class variant dictionary (sparse error matrix) learned by LRSE for each person. The facial images are from AR database [38].

It is important to note that this scenario considered here is difficult. Fig 5 shows the average recognition accuracy versus number of subjects (*p*), each of which has seven images for training. For all the methods, the performance in this scenario is not well. It is clear to see that LRSE+SC outperforms SRC, MFL, ESRC, RLRC1. When *p* = 40, LRSE+SC achieves the recognition rate at 67.17% and outperforms SRC by 12.76%. Fig 6 shows a part of dictionaries learned by MFL and LRSE when *p* = 40. We just randomly select the training samples of five persons to demonstrate. There are seven training images for each person and they are shown in Fig 6(a). Fig 6(b) presents the dictionary learned by MFL. For MFL, the dictionary is learned individually per class. Compared with the original training images, the dictionary in MFL mitigates over-fitting problem. SRC treats the original training images as dictionary. Hence, MFL performs better than SRC. The low-rank class-specific dictionaries ${\left\{D_{i}\right\}}_{i=1}^{c}$ are presented in Fig 6(c). By utilizing low-rank matrix recovery, we extract subject-specific feature that is used as supervised dictionary and separate the common feature (as shown in Fig 6(d)) that is caused by illumination, expression from the original training samples. In order to show the importance of the intra-class variant dictionary *E* for recognition, we just use the low rank class-specific dictionaries ${\left\{D_{i}\right\}}_{i=1}^{c}$ to represent the query image and then a reconstruction-based scheme for classification is adopted. This method is denoted as LR+SC. From Fig 5, it can be seen that LRSE+SC greatly outperforms LR+SC.

**Fig 7:**
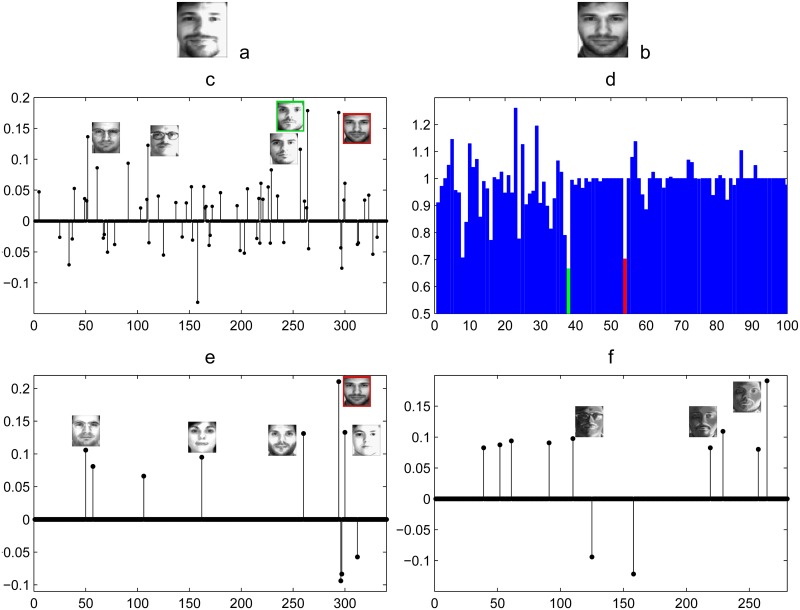
Comparison between SRC and LRSE+SC. (a) A test face image from subject 54 in the AR database: the second row is the SRC result, and the third row is the LRSE+SC result. (b) The only one training sample of subject 54. (c) Sparse coefficients associated with the original training sample dictionary. (d) Reconstruction residuals with respect to the coefficients for different classes; the red bar indicates the correct class. (e) Sparse coefficients associated with low rank supervised dictionary. (f) Sparse coefficients associated with sparse intra-class variant dictionary. Images bounded by red rectangles are the correct class, and the green rectangle ones are faces from other subjects.

The basic assumption in SRC is that there are sufficient training images of each subject and this assumption might be violated when most of subjects have only one training sample. Fig 7 shows such an example. There is only one training sample for Subject 54 as shown in Fig 7(b). Due to variant lighting, the query image (Fig 7(a)) is very similar with the training sample of Subject 38. Hence, as presented in Fig 7(c), the training sample with the largest weight is from Subject 38. The green bar in Fig 7(d) indicates that SRC fails to recognize the subject which the query image belongs to. Different from SRC, we decompose the original training samples into low-rank class-specific dictionary and sparse intra-class variant dictionary. The intra-class variant dictionary represents the illumination variations sparsely as shown in Fig 7(f). We separate the illumination and expression variations from the original training samples and the subject-specific feature is extracted. Hence, our approach mitigates over-fitting problem. As shown in Fig 7(e), the coefficient of the training sample from Subject 54 is the largest. Thus, the query image is able to be successfully recognized by LRSE+SC.

#### B. Face Recognition With Occlusion

One of the most important characteristics of SRC is its robustness to face occlusion. However, most of the current face recognition methods don’t consider that occlusions may exist in training data. When using occluded images for training, SRC might over-fitting the extreme noise of occlusion. In this subsection, we consider the training data set might contain occluded face images. There are 26 images for each subject which are taken from two separate sessions in AR database. For each session, there are seven clean images without occlusion, three images with sunglasses and three images with scarf. In our experiments, not all of subjects have occluded images for training. Firstly, we randomly select *p*(*p* = 1, 10, 20, 30, 40) classes, each of which contains occluded images. For the remaining classes, there aren’t occluded images for training. Specifically, the following three scenarios are considered.

- Sunglasses: In this scenario, for each of the *p* classes which are chosen, seven neutral images plus one image with sunglasses (randomly chosen) at Session 1 are used for training. For the remaining subjects, we just use seven neutral images at Session 1 for training. The test set contains seven images without occlusion at Session 2 and the rest of the images with sunglasses in both sessions for each subject. In total, for each of the *p* classes, we have 7 neutral images plus 5 images with sunglasses(two taken at Session 1 and three at Session 2) for testing, and for the remaining classes, there are 7 neutral images plus 6 images with sunglasses for testing.
- Scarf: We consider the training images are occluded by scarf. It’s similar to the first scenario, one image with scarf and seven neutral images at Session 1 for each of the *p* classes are randomly chosen for training. While, for other classes, only seven neutral images at Session 1 are used as training samples. The test set consists of seven neutral images without occlusion at Session 2 and the remaining images with scarf in both sessions for each subject.
- Sunglasses+scarf: The third scenario is that two corrupted images (one with sunglasses and one with scarf) for each of the *p* subjects are randomly selected from the Session 1 and used for training. And seven neutral images at Session 1 for each of the 100 subjects for training. seven images without occlusion at Session 2 and all the images with scarf and sunglasses which aren’t selected for training in both sessions for each subject are used for testing.

**Fig 8:**
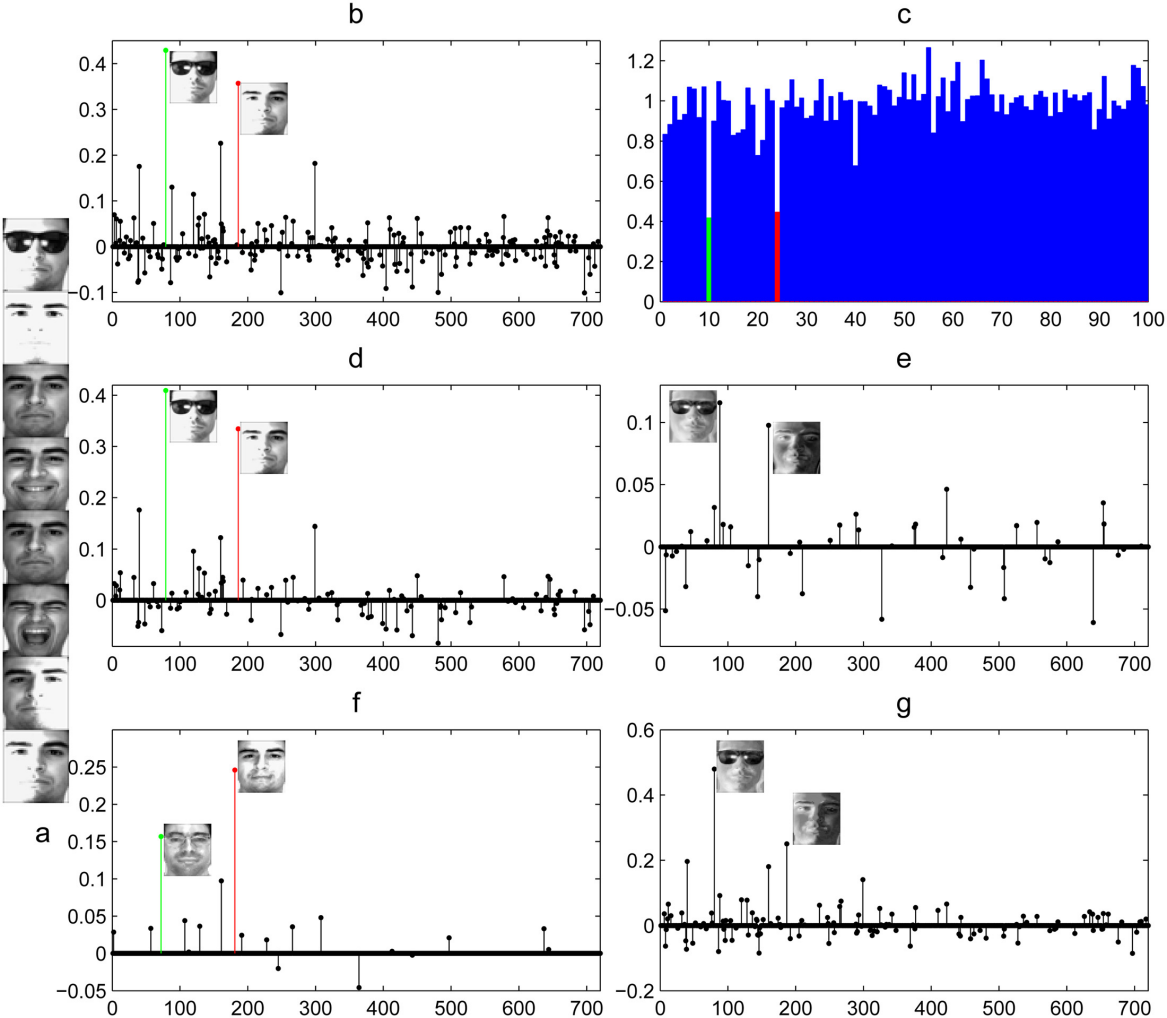
Example of the sparse representation of a query image with disguise (100 subjects). (a) The images from subject 24 in the AR database: the first row is the query image, and the remaining images are training samples of subject 24. (b) The values of the sparse coefficients recovered from SRC are plotted with together the two bases that correspond to the two largest sparse coefficients. (c) Reconstruction residuals with respect to the coefficients for different classes; the red bar indicates the correct class. (d),(e) Illuminate the coefficients solved by ESRC. The sparse coefficients associated with the training sample dictionary and intra-class variant dictionary are plotted in (d),(e), respectively. The coefficients solved by LRSE+SC are shown in (f),(g). (f) Sparse coefficients associated with low rank supervised dictionary. (g) Sparse coefficients associated with sparse intra-class variant dictionary. Red (darker) entry correspond to the base belonging to subject 24 and green (darker) entry correspond to the base belonging to subject 10.

In all three scenarios, some subjects have occluded images for training, but the rest of subjects don’t have. Hence, the occluded regions might be regarded as subject-specific feature for SRC. The experiments are repeated for 5 times and recognition accuracies are averaged. Tables 1, 2 and 3 list the results for sunglasses, scarf and sunglasses+scarf respectively. It’s clear to see that LRSE+SC outperforms SRC, ESRC, MFL and RLRC1. For example, when *p* = 10, we achieve recognition rates at 75.2%, 81.51% and 73.64% for the scenarios of sunglasses, scarf and sunglass+scarf, respectively, while the recognition rates of SRC are 71.11%, 78.29% and 67.81%. From these three tables, both RLRC1 and MFL perform poorly, since these two methods can not separate the occlusion information from the original images. ESRC and LRSE+SC introduce an intra-class variant dictionary to represent the variations between the query image and the training images. Fig 8 shows the sparse coding of a query image with sunglass. The query image comes from Subject 24 and is presented in the first row of Fig 8(a). The remaining seven unoccluded images of Fig 8(a) are training samples of Subject 24. There is one image from Subject 10 in the training set, which is potentially occluded by sunglass. Due to occlusion, the occluded image of Subject 10 looks like the query image. From Fig 8(b), the solution recovered by SRC is not sparse and the largest coefficient corresponds to the occluded image of Subject 10. Fig 8(c) shows the corresponding residuals with respect to the 100 subjects. The green bar indicates that SRC fails to identify the subject. In order to deal with occlusion, ESRC uses an intra-class variant dictionary to represent the possible variations. Fig 8(d) and 8(e) plot the coefficients correspond to the training sample dictionary and intra-class variant dictionary. However, ESRC can not separate the occluded part from the query image. Therefore, the value of the coefficient corresponding to the image with sunglass is also the largest. Different from ESRC, LRSE+SC decomposes the original training images into low-rank supervised dictionary and sparse intra-class variant dictionary. Hence, the low-rank supervised dictionary doesn’t contain occlusion variations and the value of the coefficient corresponding to the base from Subject 24 is the largest, as presented in Fig 8(f) and 8(g). LRSE+SC identifies the subject correctly.

**Table 1:** Face recognition rates of different methods on a subset of the AR database. In this experiment, the training set is corrupted by face images with sunglasses.

Approach	Recognition Rate(%)				
	*p* = 1	*p* = 10	*p* = 20	*p* = 30	*p* = 40
SRC	64.63	71.11	74.95	76.62	79.19
ESRC	66.5	73.11	76.56	78.03	80.3
RLRC1	53.53	57.64	64.1	66.01	67.39
RLRC2	48.62	48.51	51.6	54.06	56.92
MFL	63	68.04	71.78	72.85	74.53
LR+SC	39.6	40.75	41.39	42.34	42.71
LRSE+SC	65.97	75.2	78.96	81.22	82.95

**Table 2:** Face recognition rates of different methods on a subset of the AR database. In this experiment, the training set is corrupted by face images with scarf.

Approach	Recognition Rate(%)				
	*p* = 1	*p* = 10	*p* = 20	*p* = 30	*p* = 40
SRC	72.8	78.29	79.7	80.58	82.09
ESRC	73.26	79.41	80.84	81.63	83.01
RLRC1	62.95	62.51	66.17	68.31	70.87
RLRC2	46.82	47.65	50.04	52.36	54.36
MFL	68.99	73.81	76.17	76.36	77.46
LR+SC	44.14	49.19	46.32	50.86	52.53
LRSE+SC	75.04	81.51	83.17	83.95	84.9

**Table 3:** Face recognition rates of different methods on a subset of the AR database. In this experiment, the training set is corrupted by face images with sunglasses and scarf.

Approach	Recognition Rate(%)				
	*p* = 1	*p* = 10	*p* = 20	*p* = 30	*p* = 40
SRC	60.87	67.81	72.41	74.28	76.32
ESRC	62.66	70.31	74.06	75.58	77.53
RLRC1	45.92	47.29	52.12	56.45	59.82
RLRC2	35.59	35.48	38.68	42.31	45.27
MFL	47.92	65.01	67.58	69.05	71.91
LR+SC	34.49	38.75	36.26	38.46	38.14
LRSE+SC	61.59	73.64	77.06	78.69	80.35

#### C. Face Recognition With Random Faces

Recently, Random Projection [39] has emerged as a powerful tool in dimensionality reduction. In order to evaluate the performance of the LRSE+SC algorithm on different feature extraction methods, we will use random faces as feature descriptors in AR face database for recognition. The dataset used in our experiment is provided by Jiang [40] and it’s a subset of AR database which consists of 2,600 images from 50 female subjects and 50 male subjects. Each image is projected onto a 540-dimensional subspace with a randomly generated matrix from a zero-mean normal distribution. Each row of the random matrix is unit length. For each subject, *n*(*n* = 4, 5, 6, 7, 8) images are randomly selected for training and the other images(26 − *n*) for testing. All experiments run 20 times and the average recognition rates of different methods and their corresponding standard deviations are shown in Table 4.

**Table 4:** Mean recognition rates(%) and standard deviations of different methods on AR database.

Algorithm	4 train	5 train	6 train	7 train	8 train
SRC	81.23±0.89	85.53±0.91	88.73±0.92	91.02±0.73	92.79±0.51
ESRC	82.09±0.78	86.16±0.86	89.26±0.77	91.57±0.71	93.13±0.52
LRC	37.44±1.06	44.82±1.14	52.44±1.29	58.65±1.48	64.52±1.57
RLRC1	70.86±1.13	74.06±1.43	77.49±1.11	80.45±1.69	82.79±1.56
RLRC2	48.95±0.91	56.46±1.25	63.61±1.27	69.36±1.60	74.21±1.55
MFL	78.52±0.91	83.15±1.00	86.59±1.03	89.37±0.87	91.15±0.79
LRSE+SC	83.45±0.82	87.93±0.80	90.62±0.71	92.83±0.63	94.15±0.66

It’s clear that the proposed LRSE+SC consistently outperforms others. Since we have small number of training data per class, the recognition rates of LRC is relatively low. In order to overcome the issue of small sample size, RLRC1 and RLRC2 are proposed. The basic idea behind these two methods is that the basis vectors of each class-specific subspace are composed of the class-specific basis vectors owned by one class only and the common basis vectors shared by many classes. From Table 4, the recognition rates of RLRC1 are significantly higher than the recognition rates of LRC. MFL independently learns dictionary per class and some useful information may be loss in this procedure. Therefore, the performance of MFL may not be better than SRC. ESRC and LRSE+SC utilize the intra-class variations, such as lighting changes, and these two methods achieve good performance. However, comparing with ESRC, LRSE+SC can separate intra-class variations from original training samples.

### FERET Database

The FERET database [41] is one of the most widely used databases for face recognition. A subset of the FERET database is used to test the performance of the LRSE+SC, in which there are 720 images of 120 subjects (each subject containing six images). Images with different illuminations and expressions are collected in this subset. All the images are cropped with dimension 44 × 40.

**Fig 9:**
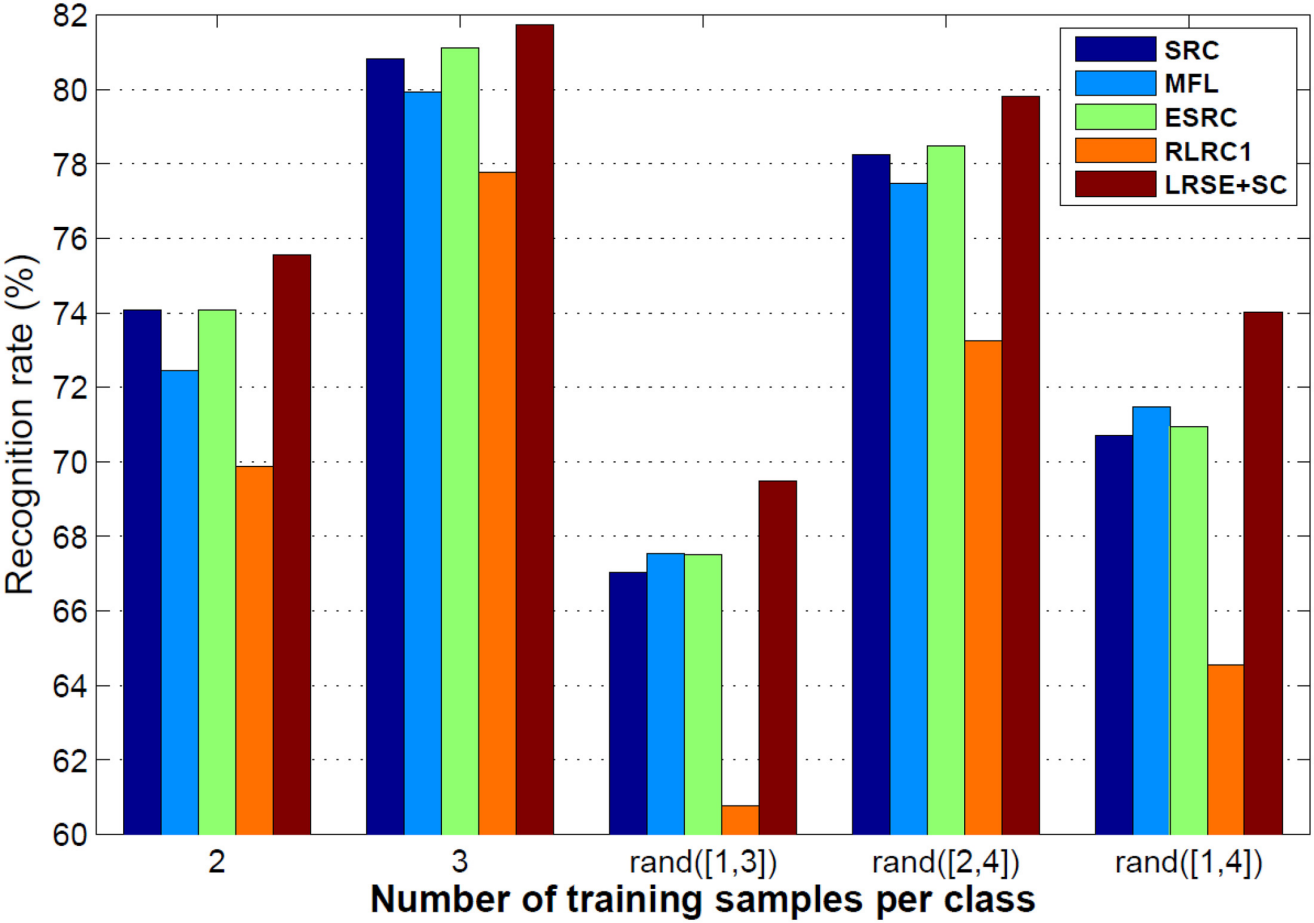
Recognition rate versus training sample number on FERET database. *rand*([1, 3]) is a random number between 1 and 3.

In this experiment, for each subject, a few images are selected as training samples. We also consider the situation that the number of training samples for each subject is unequal. Hence, five groups experiments are designed. 2, 3, *rand*([1, 3]), *rand*([2, 4]), *rand*([1, 4]) images of each subject are chosen for training, respectively, and the remaining images are used as test samples. we select images randomly and repeat for 5 times in each condition. The average recognition rates across 5 runs of each method are presented in Fig 9. LRSE+SC achieves the highest recognition rates in the five experiments, which demonstrates its capability to deal with the small sample size problem. Since SRC and MFL need sufficient training samples, they do not work well. As we know, when the number of training samples per subject is not equal, LRSE+SC mitigates the over-fitting problem and performs better than other methods.

### FRGC Database

The FRGC v2.0 data [42] is used for evaluating the real processing and recognition performance, which is a large-scale face database designed with uncontrolled indoor and outdoor setting. In this experiment, an outdoor lighting set is exploited as unstrained face recognition benchmark. The outdoor lighting subset contains 275 subjects, and each subject has five uncontrolled lighting images. They are cropped and normalized to 50 × 50. The uncontrolled images are taken in varying illumination conditions, e.g., hallways, atriums, or outside. We randomly select *n*(*n* = 2, 3) images per person for training and the remained for testing, and the experiment is repeated for 20 times.

The average accuracies and standard deviations are listed in Table 5. We can see that LRSE+SC outperforms all the other methods. When the number of training samples per class is two, LRSE+SC obtains the top results of 85.88±0.87, which is followed by the ESRC approach. It can be also be seen that the recognition rates of SRC are almost the same as ESRC.

**Table 5:** Mean recognition rates(%) and standard deviations of different methods on FRGC database.

Algorithm	2 train	3 train
SRC	81.78±0.98	89.81±1.32
ESRC	81.81±0.97	90.17±1.31
LRC	52.68±1.41	61.32±1.74
RLRC1	75.10±1.23	82.53±1.44
RLRC2	59.58±1.07	69.11±1.42
MFL	79.20±0.84	87.47±1.25
LRSE+SC	85.88±0.87	92.93±0.98

### LFW Database

In this section, we test the effectiveness of LRSE+SC in handling the problem of unconstrained face recognition. The LFW database [43] consists of images of 5,749 individuals captured from uncontrolled environment. Same as [44], a subset of aligned LFW [45] is chosen for testing, which includes 143 subjects with no less than 11 samples per subject. For each subject, the first 10 images are used as training samples and the remaining images are used for testing. In order to represent the face image, Gabor magnitude [46] feature and Local Binary Pattern (LBP) [47] feature are extracted. For each image, it is partitioned into 2 × 2 blocks; and then the discrimination-enhanced feature is obtained by performing LDA in each block; finally, the features of all blocks are concatenated and the feature dimension is 560. It can be seen from Table 6 that LRSE+SC achieves the best performance.

**Table 6:** Face recognition rates of competing methods on the LFW database with Gabor feature and LBP feature.

Feature	Recognition Rate(%)						
	SVM	MFL	RLRC1	RLRC2	SRC	ESRC	LRSE+SC
Gabor	42.35	68.91	61.66	61.66	68.66	70.55	71.10
LBP	18.48	65.63	56.05	56.71	61.63	66.07	66.98

### Statistical Evaluation

In order to find significant differences in performance across the six different classifiers (e.g. SRC, ESRC, MFL, RLRC1, RLRC2 and LRSE+SC), rank based statistics are employed. According to [48], we use the Friedman test with the corresponding post-hoc test for statistical comparisons of the six different classifiers (e.g. SRC, ESRC, MFL, RLRC1 and LRSE+SC).

Firstly, the Friedman test is employed to assess whether the average ranks of different approaches are statistically different from the mean rank. This test ranks the methods according to their results for each dataset separately, thus the best performing algorithm gets rank 1, the second best one gets rank 2, etc. Since six approaches are used for comparison, the mean rank is (1 + ⋯ + 6)/6 = 3.5. Then, the Friedman test compares the average ranks of methods. Under the null-hypothesis, which states that the algorithms are equivalent and their ranks should be equal to rank 3.5, we calculate the following refined Friedman statistic *F*_*F*_ with six algorithms and 22 test sets:

$$\begin{array}{c} \begin{array}{cc} \chi_{F}^{2} & =\frac{12\times 22}{6\times (6+1)}\left[3.17^{2}+2.16^{2}+4.09^{2}+4.7^{2}+5.93^{2}+1.05^{2}-\frac{6\times 7^{2}}{4}\right]=98.53\\ F_{F} & =\frac{21\times 98.53}{22\times 5-98.53}=180.48∼F\left(6-1,22\times \left(6-1\right)\right). \end{array} \end{array}$$ (15)

The critical value of *F*(5, 105) at *α* = 0.05 is 2.3. Since *F*_*F*_ > 2.3, the null-hypothesis is rejected, which means that the performance of different approaches is statistically significant difference.

Therefore, the next step is the post-hoc test to compare the proposed method with others. Under this test, the critical difference (*CD*) is used to measure whether the performance of two algorithms is significantly different with each other in terms of the corresponding average ranks. The critical difference is defined as follows:

$$\begin{array}{c} \begin{array}{c} CD=q_{\alpha }\;\sqrt{\frac{6\times (6+1)}{6\times 22}}=0.5641q_{\alpha }, \end{array} \end{array}$$ (16)

where *q*_*α*_ are critical values and given in [48].

For our results, *CD* = 2.576 × 0.5641 = 1.4531 at *α* = 0.05. Table 7 lists the differences between the average ranks of LRSE+SC and the other methods. It’s clear that the proposed LRSE+SC method is better than SRC, MFL, RLRC1 and RLRC2 at *α* = 0.05, since the differences in average ranks are larger than the *CD* = 1.45. Due to the intra-class variant dictionary, ESRC can alleviate the small sample size problem and obtain better performance than SRC. However, for significance level *α* = 0.05, we can’t say LRSE+SC is significantly different from ESRC.

**Table 7:** Differences between the average ranks of the six algorithms and the average rank of LRSE+SC.

Algorithm	SRC	ESRC	MFL	RLRC1	RLRC2	LRSE+SC
Average Rank	3.07	2.16	4.09	4.7	5.93	1.05
Difference	2.02	1.11	3.04	3.65	4.88	

### Parameter Selection

There are two parameters, *λ*_1_ and *λ*_2_ in our method LRSE+SC. The two parameters have very clear physical meaning, which could guide the setting of these parameters. The parameter *λ*_1_ trades off the rank of *D*_*i*_ versus the sparsity of the error *E*_*i*_. According to the theoretical considerations in [19], the correct scaling is *λ*_1_ = *O*(*m* ^−1/2^). For example, if the dimension of feature vector is *m* = 1760, we find the optimal *λ*_1_ in the neighborhood of $\frac{1}{\sqrt{m}}=0.023$. The parameter *λ*_2_ balances the tradeoff between the sparsity of representation and reconstruction error. We find a good value in the ranges of *λ*_2_ = [10^−4^, 10^−1^].

**Fig 10:**
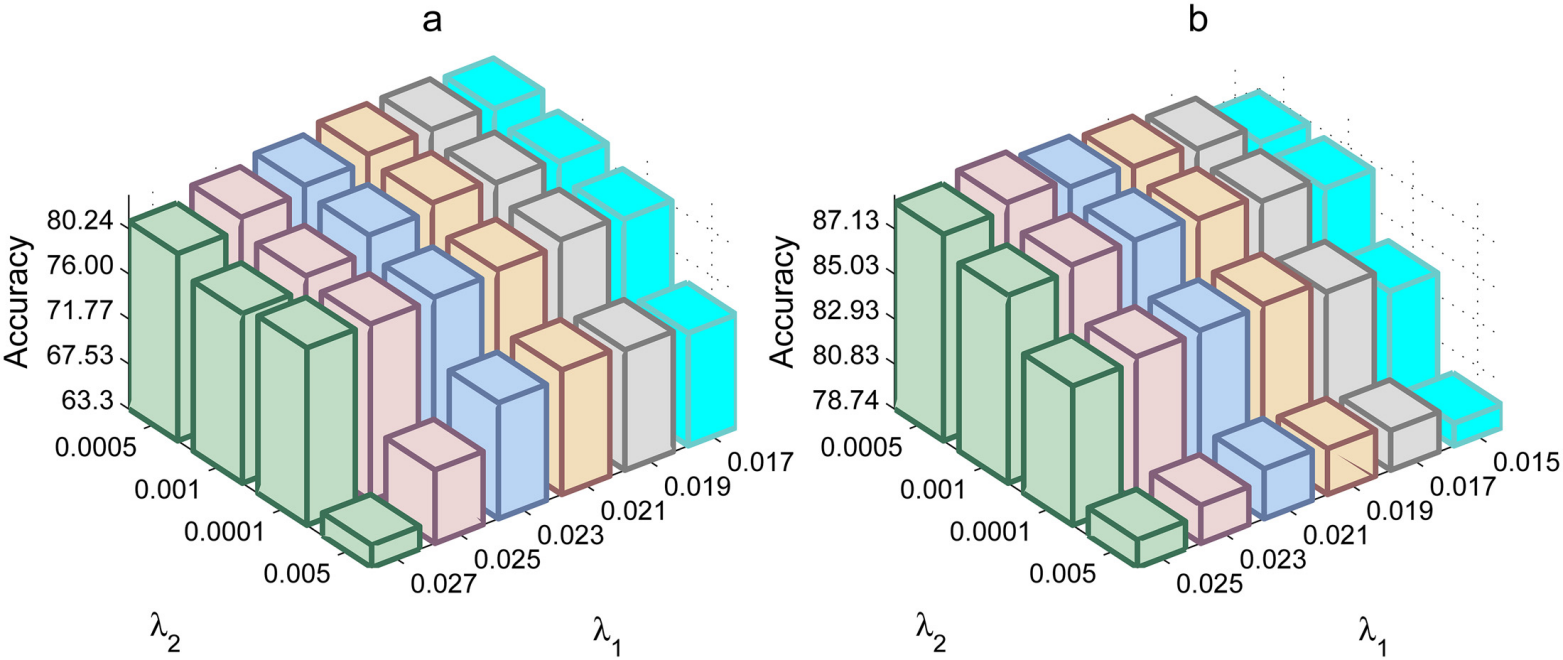
Effects of parameter selection of *λ*_1_ and *λ*_2_ on the classification accuracy on AR database. (a) The accuracy with varying parameters on face recognition without occlusion. (b) The accuracy with varying parameters on face recognition with occlusion.

In order to study the influences of parameters *λ*_1_, *λ*_2_ over recognition accuracy, we perform two experiments on AR database. Fig 10 shows the evaluation results of LRSE+SC with different values of *λ*_1_, *λ*_2_. These two examples demonstrate that the optimal values of *λ*_1_, *λ*_2_ are correlated with training samples and test samples. However, when *λ*_2_ = 0.001, 0.0005, LRSE+SC achieves good performance. It is easy to see that LRSE+SC can achieve good performance when the value of *λ*_1_ in the neighborhood of 0.023.

## Conclusions

In this paper, we have introduced a novel face recognition framework by utilizing low rank matrix recovery. In this mechanism, we try to decompose the original images into the class-specific feature and within-class variant feature. We have demonstrated theoretically and experimentally that the within-class variant feature which is separated from the original images is very important to deal with small sample size problem.

Our method leverages low-rank and sparse error matrix decomposition technique and sparse representation scheme. Firstly, we recover the low-rank matrix of each subject by removing the sparse error in the training images. Then the low-rank matrix can be used as supervised dictionary to coding the test samples. Our algorithm regards the sparse error matrix of all subjects as sparse within-class variant dictionary to represent the variants of the test image and training images from the same class, which may be caused by illuminations, expressions and disguises. Experiments confirm that LRSE+SC approach outperforms SRC and ESRC for small sample size problem.

## References

[1] Turk M, Pentland A. Eigenfaces for recognition. Journal of Cognitive Neuroscience. 1991;3(1):71–86. 23964806 10.1162/jocn.1991.3.1.71

[2] Belhumeur PN, Hespanha JP, Kriegman DJ. Eigenfaces vs. fisherfaces: Recognition using class specific linear projection. IEEE Transactions on Pattern Analysis and Machine Intelligence. 1997;19(7):711–720. 10.1109/34.598228

[3] He X, Yan S, Hu Y, Niyogi P, Zhang HJ. Face recognition using laplacianfaces. IEEE Transactions on Pattern Analysis and Machine Intelligence. 2005;27(3):328–340. 10.1109/TPAMI.2005.55 15747789

[4] Wang Y, Zhang L, Liu Z, Hua G, Wen Z, Zhang Z, et al Face relighting from a single image under arbitrary unknown lighting conditions. IEEE Transactions on Pattern Analysis and Machine Intelligence. 2009;31(11):1968–1984. 10.1109/TPAMI.2008.244 19762925

[5] Fidler S, Skocaj D, Leonardis A. Combining reconstructive and discriminative subspace methods for robust classification and regression by subsampling. IEEE Transactions on Pattern Analysis and Machine Intelligence. 2006;28(3):337–350. 10.1109/TPAMI.2006.46 16526421

[6] Chen L, Liao H, Ko M, Lin J, Yu G. A new LDA-based face recognition system which can solve the small sample size problem. Pattern Recognition. 2000;33(10):1713–1726. 10.1016/S0031-3203(99)00139-9

[7] Wright J, Yang A, Ganesh A, Sastry S, Ma Y. Robust face recognition via sparse representation. IEEE Transactions on Pattern Analysis and Machine Intelligence. 2009;31(2):210–227. 10.1109/TPAMI.2008.79 19110489

[8] Yang M, Zhang L, Yang J, Zhang D. Metaface learning for sparse representation based face recognition. In: Proceedings of IEEE International Conference on Image Processing; 2010. p. 1601–1604.

[9] Mairal J, Bach F, Ponce J, Sapiro G, Zisserman A. Discriminative learned dictionaries for local image analysis. In: Proceedings of IEEE Conference on Computer Vision and Pattern Recognition; 2008. p. 1–8.

[10] Pham D, Venkatesh S. Joint learning and dictionary construction for pattern recognition. In: Proceedings of IEEE Conference on Computer Vision and Pattern Recognition; 2008. p. 1–8.

[11] Ramirez I, Sprechmann P, Sapiro G. Classification and clustering via dictionary learning with structured incoherence and shared features. In: Proceedings of IEEE Conference on Computer Vision and Pattern Recognition; 2010. p. 3501–3508.

[12] Zhang Q, Li B. Discriminative K-SVD for dictionary learning in face recognition. In: Proceedings of IEEE Conference on Computer Vision and Pattern Recognition; 2010. p. 2691–2698.

[13] Yang M, Zhang L, Feng X, Zhang D. Fisher Discrimination Dictionary Learning for Sparse Representation. In: Proceedings of IEEE International Conference on Computer Vision; 2011. p. 543–550.

[14] Jiang Z, Lin Z, Davis L. Learning a discriminative dictionary for sparse coding via label consistent K-SVD. In: Proceedings of IEEE Conference on Computer Vision and Pattern Recognition; 2011. p. 1697–1704.

[15] Zhu G, Yan S, Ma Y. Image tag refinement towards low-rank, content-tag prior and error sparsity. In: Proceedings of the International Conference on Multimedia; 2010. p. 461–470.

[16] Min K, Zhang Z, Wright J, Ma Y. Decomposing background topics from keywords by principal component pursuit. In: Proceedings of ACM International Conference on Information and Knowledge Management; 2010. p. 269–278.

[17] Shen X, Wu Y. A unified approach to salient object detection via low rank matrix recovery. In: Proceedings of IEEE Conference on Computer Vision and Pattern Recognition; 2012. p. 853–860.

[18] Zhang Z, Ganesh A, Liang X, Ma Y. TILT: transform invariant low-rank textures. International Journal of Computer Vision. 2012;99(1):1–24. 10.1007/s11263-012-0515-x

[19] Candès E, Li X, Ma Y, Wright J. Robust principal component analysis? Journal of the ACM. 2011;58(3):11.

[20] Ma L, Wang C, Xiao B, Wen Z. Sparse representation for face recognition based on discriminative low-rank dictionary learning. In: Proceedings of IEEE Conference on Computer Vision and Pattern Recognition; 2012. p. 2586–2593.

[21] Chen CF, Wei CP, Wang YCF. Low-rank matrix recovery with structural incoherence for robust face recognition. In: Proceedings of IEEE Conference on Computer Vision and Pattern Recognition; 2012. p. 2618–2625.

[22] Zhang Y, Jiang Z, Davis LS. Learning Structured Low-rank Representations for Image Classification. In: Proceedings of IEEE Conference on Computer Vision and Pattern Recognition; 2013. p. 676–683.

[23] Lu J, Plataniotis KN, Venetsanopoulos AN. Face recognition using LDA-based algorithms. IEEE Transactions on Neural Networks. 2003;14(1):195–200. 10.1109/TNN.2002.806647 18238001

[24] Wagner A, Wright J, Ganesh A, Zhou Z, Mobahi H, Ma Y. Toward a practical face recognition system: Robust alignment and illumination by sparse representation. IEEE Transactions on Pattern Analysis and Machine Intelligence. 2012;34(2):372–386. 10.1109/TPAMI.2011.112 21646680

[25] Deng W, Hu J, Guo J. Extended SRC: Undersampled Face Recognition via Intra-Class Variant Dictionary. IEEE Transactions on Pattern Analysis and Machine Intelligence. 2012;34(9):1864–1870. 10.1109/TPAMI.2012.30 22813959

[26] Wei C, Wang Y. Learning auxiliary dictionaries for undersampled face recognition. In: Proceedings of IEEE International Conference on Multimedia and Expo; 2013. p. 1–6.

[27] Mi J, Liu J, Wen J. New Robust Face Recognition Methods Based on Linear Regression. PLoS ONE. 2012;7(8):e42461 10.1371/journal.pone.0042461 22879992 PMC3413675

[28] Chien JT, Wu CC. Discriminant waveletfaces and nearest feature classifiers for face recognition. IEEE Transactions on Pattern Analysis and Machine Intelligence. 2002;24(12):1644–1649. 10.1109/TPAMI.2002.1114855

[29] Naseem I, Togneri R, Bennamoun M. Linear regression for face recognition. IEEE Transactions on Pattern Analysis and Machine Intelligence. 2010;32(11):2106–2112. 10.1109/TPAMI.2010.128 20603520

[30] Torre FDL, Black MJ. Robust principal component analysis for computer vision. In: Proceedings of IEEE International Conference on Computer Vision; 2001. p. 362–369.

[31] Torre FDL, Black MJ. A framework for robust subspace learning. Proceedings of International Journal of Computer Vision. 2003;54(1):117–142. 10.1023/A:1023709501986

[32] Ke Q, Kanade T. Robust l1 norm factorization in the presence of outliers and missing data by alternative convex programming. In: Proceedings of IEEE Conference on Computer Vision and Pattern Recognition; 2005. p. 739–746.

[33] Lin Z, Chen M, Ma Y. The augmented lagrange multiplier method for exact recovery of corrupted low-rank matrices. arXiv preprint arXiv:10095055. 2010;.

[34] Beck A, Teboulle M. A fast iterative shrinkage-thresholding algorithm for linear inverse problems. SIAM Journal on Imaging Sciences. 2009;2(1):183–202. 10.1137/080716542

[35] Lee H, Battle A, Raina R, Ng A. Efficient sparse coding algorithms. In: Advances in Neural Information Processing Systems; 2006 p. 801–808.

[36] Kim SJ, Koh K, Lustig M, Boyd S, Gorinevsky D. An interior-point method for large-scale l1-regularized least squares. IEEE Journal of Selected Topics in Signal Processing. 2007;1(4):606–617. 10.1109/JSTSP.2007.910971

[37] Malioutov DM, Cetin M, Willsky AS. Homotopy continuation for sparse signal representation. In: Proceedings of IEEE International Conference on Acoustics, Speech, and Signal Processing; 2005.

[38] Martinez A, Benavente R. The AR face database. Computer Vision Center, Technical Report. 1998;24.

[39] Baraniuk R, Wakin M. Random projections of smooth manifolds. Foundations of Computational Mathematics. 2009;9(1):51–77. 10.1007/s10208-007-9011-z

[40] Jiang Z, Lin Z, Davis L. Label consistent K-SVD: learning a discriminative dictionary for recognition. IEEE Transactions on Pattern Analysis and Machine Intelligence. 2013;35(11):2651–2664. 10.1109/TPAMI.2013.88 24051726

[41] Phillips P, Moon H, Rizvi S, Rauss P. The FERET evaluation methodology for face-recognition algorithms. IEEE Transactions on Pattern Analysis and Machine Intelligence. 2000;22(10):1090–1104. 10.1109/34.879790

[42] Phillips P, Flynn P, Scruggs T, Bowyer K, Chang J, Hoffman K, et al. Overview of the face recognition grand challenge. In: Proceedings of IEEE Conference on Computer Vision and Pattern Recognition; 2005. p. 947–954.

[43] Huang G, Ramesh M, Berg T, Learned-Miler E. Labeled faces in the wild: A database for studying face recognition in unconstrained environments Technical Report 07-49, University of Massachusetts, Amherst; 2007.

[44] Yang M, Zhang L, Zhang D, Wang S. Relaxed collaborative representation for pattern classification. In: Proceedings of IEEE Conference on Computer Vision and Pattern Recognition; 2012. p. 2224–2231.

[45] Taigman Y, Wolf L, Hassner T. Multiple one-shots for utilizing class label information. In: Proceedings of British Machine Vision Coference; 2009. p. 1–12.

[46] Liu C, Wechsler H. Gabor feature based classification using the enhanced fisher linear discriminant model for face recognition. IEEE Transactions on Image Processing. 2002;11(4):467–476. 10.1109/TIP.2002.999679 18244647

[47] Ahonen T, Hadid A, Pietikainen M. Face description with local binary patterns: Application to face recognition. IEEE Transactions on Pattern Analysis and Machine Intelligence. 2006;28(12):2037–2041. 10.1109/TPAMI.2006.244 17108377

[48] Demšar J. Statistical comparisons of classifiers over multiple data sets. Journal of Machine Learning Research. 2006;7:1–30.

